# An economic evaluation of an integrated care pathway in geriatric rehabilitation for older patients with complex health problems

**DOI:** 10.1371/journal.pone.0191851

**Published:** 2018-02-28

**Authors:** Irma H. J. Everink, Jolanda C. M. van Haastregt, Silvia M. A. A. Evers, Gertrudis I. J. M. Kempen, Jos M. G. A. Schols

**Affiliations:** 1 Department of Health Services Research and Care and Public Health Research Institute (CAPHRI), Faculty of Health, Medicine and Life Sciences, Maastricht University, Maastricht, The Netherlands; 2 Trimbos Institute, Netherlands Institute of Mental Health and Addiction, Centre for Economic Evaluations, Utrecht, the Netherlands; 3 Department of Family Medicine and Care and Public Health Research Institute (CAPHRI), Faculty of Health, Medicine and Life Sciences, Maastricht University, Maastricht, The Netherlands; University of Glasgow, UNITED KINGDOM

## Abstract

**Background:**

Integrated care pathways which cover multiple care settings are increasingly used as a tool to structure care, enhance coordination and improve transitions between care settings. However, little is known about their economic impact. The objective of this study is to determine the cost-effectiveness and cost-utility of an integrated care pathway designed for patients with complex health problems transferring from the hospital, a geriatric rehabilitation facility and primary care.

**Methods:**

This economic evaluation was performed from a societal perspective alongside a prospective cohort study with two cohorts of patients. The care as usual cohort was included before implementation of the pathway and the care pathway cohort after implementation of the pathway. Both cohorts were measured over nine months, during which intervention costs, healthcare costs, patient and family costs were identified. The outcome measures were dependence in activities of daily living (measured with the KATZ-15) and quality adjusted life years (EQ-5D-3L). Costs and effects were bootstrapped and various sensitivity analyses were performed to assess robustness of the results.

**Results:**

After nine months, the average societal costs were significantly lower for patients in the care pathway cohort (€50,791) versus patients in the care as usual cohort (€62,170; CI = -22,090, -988). Patients in the care pathway cohort had better scores on the KATZ-15 (1.04), indicating cost-effectiveness. No significant differences were found between the two groups on QALY scores (0.01).

**Conclusions:**

The results of this study indicate that the integrated care pathway is a cost-effective intervention. Therefore, dissemination of the integrated care pathway on a wider scale could be considered. This would provide us the opportunity to confirm the findings of our study in larger economic evaluations. When looking at QALYs, no effects were found. Therefore, it is also recommended to explore if therapy in geriatric rehabilitation could also pay attention to other quality of life-related domains, such as mood and social participation.

## Background

It is not always possible for community-dwelling older patients who are admitted to the hospital to directly return home after discharge. This population will often experience functional decline and deterioration in self-care abilities, usually caused by their acute condition and inactivity during hospital stays that warrant admission to an inpatient geriatric rehabilitation facility.[1] In these facilities, patients receive multidisciplinary care to restore functional independence and mobility and prepare patients to safely return to their original home situation.[2, 3] Because these community-dwelling older patients require care from different healthcare providers in various settings, they need to make multiple transitions between care settings. These care transitions expose patients to problems regarding continuity of care, such as lack of communication between care providers, errors in medication lists, or insufficient quality of discharge summaries.[4–6] When continuity of care is not adequately organized, serious negative consequences may occur, such as deterioration of illness, hospital readmissions, permanent placement in nursing homes, or even death.[4, 7, 8] Not only do these adverse events cause considerable harm to patients and their informal caregivers, these adverse events also incur high costs. For example, in the U.S. nearly 20% of all older adults admitted to the hospital are readmitted within a month, costing approximately $25 billion every year. It is estimated that of these 20% readmissions, 75% could be prevented.[9] Furthermore, medication errors are estimated to cost $3.5 billion annually in the U.S. Two-thirds of the medication errors occur during care transitions.[10, 11]

Intersectoral integrated care pathways are increasingly used as a tool to improve care transitions. Research showed positive results of care pathways including the hospital setting and primary care, on morbidity, drug-related adverse events, hospital readmission rates, emergency department visits and care coordination. [12–16] Integrated care pathways describe the sequence and timing of actions in order to achieve patient outcomes with optimal efficiency. They are intended to structure care and enhance coordination with the goal of improved efficiency, patient safety and continuity of care.[17–19] A systematic review of the literature by Allen and colleagues[15] demonstrated that integrated care pathways are effective in improving communication with patients, informal caregivers and health professionals, and in ensuring that patients receive safe and relevant interventions or assessments.[20] Although different systematic reviews have yielded positive effects for care pathways[20–22], less is known about their economic impact.[23, 24] A systematic review by van Herck and colleagues published in 2004 focused on the identification of indicators to evaluate clinical pathways. This review found that of the 131 papers comprising any form of financial evaluation, more than 80% reported a positive effect.[21] However, it was unclear from these studies which methodology was used to calculate costs and which costs, in which settings, were taken into account. In more recent years, only a few studies have assessed the cost-effectiveness of integrated care pathways, and among these studies, the patient groups and settings where the pathways were implemented, vary widely.[25–29]

Between 2012 and 2014, an integrated care pathway in geriatric rehabilitation was developed and implemented in the Netherlands for older patients with complex health problems.[30] In Dutch geriatric rehabilitation facilities, patients are categorized into four groups: 1) patients with strokes; 2) trauma orthopedics; 3) elective orthopedics; and 4) the remaining, classified as patients with complex health problems. Patients with complex health problems often have multi-morbidities and may also have mild cognitive impairments and/or behavioral problems. However, patients with severe cognitive impairments (i.e. persons with advanced dementia) are in general not admitted to geriatric rehabilitation in the Netherlands, because they do not have sufficient cognitive skills to participate in a rehabilitation program.

This pathway aimed to improve continuity and coordination of care for community-dwelling patients who go through the trajectory of hospitalization, admission to a post-acute geriatric rehabilitation facility and discharge back to the home situation where they often receive primary care (such as care from a GP, home care and physiotherapy). The pathway focussed on improving communication, triage and transfers of patients between the hospital, the geriatric rehabilitation facility and primary care organizations.[30] As patients who go through this pathway use multiple healthcare services, the corresponding costs may be high. Implementing the integrated care pathway was expected to result in decreased dependence in activities of daily living, improved quality of life and reduced overall costs. The objective of this study was to determine the cost-effectiveness of this integrated care pathway from a societal perspective by comparing a cohort of patients who received care as usual with a cohort of patients who received care in the integrated care pathway.

## Methods

### Guidelines

This study followed the Consolidated Health Economic Evaluation Reporting Standards (CHEERS) Statement[31] and the Dutch manual for cost research and reference prices in health care.[30] The study design and methods were approved by the independent Medical Ethics Committee of University Hospital Maastricht/Maastricht University (#11-4-020).

### Study design

This study describes an economic evaluation from a societal perspective. This evaluation was embedded in a prospective cohort study with two cohorts of patients and informal caregivers. The design, methods, and effects of this prospective cohort study will be published elsewhere. The two cohorts of patients and informal caregivers were recruited in the geriatric rehabilitation facility where the pathway was implemented. This facility was located in Maastricht (the South of the Netherlands). The first cohort (the reference cohort) received care as usual and was included between April 2011—March 2012, prior to implementation of the integrated care pathway. In 2013, the integrated care pathway was implemented. The second cohort (the care pathway cohort) was included between April 2013 and September 2014, after implementation of the integrated care pathway. This study design and methods were approved by the Medical Ethics Committee of University Hospital Maastricht (#11-4-020).

### Setting and subjects

The participants of this study were patients who were admitted to a geriatric rehabilitation facility (which in the Netherlands are usually situated in a nursing home) and their informal caregivers. These patients were eligible for participation if they were part of the group of patients with complex health problems, were admitted to the geriatric rehabilitation facility in the inclusion period mentioned in the paragraph ‘study design’, aged ≥ 65 years, were community-dwelling and hospitalized prior to admission to the geriatric rehabilitation facility. Patients were excluded if the elderly care physician assessed their cognitive status as insufficient to participate in the study. If a patient confirmed having an informal caregiver, this informal caregiver was invited to participate in the study as well. The informal caregiver could be a family member or a non-family member, provided that they delivered voluntary and unpaid care on a structural base (e.g. for a longer period of time). All patients and informal caregivers provided written informed consent prior to participating in the data collection.

### Intervention

The integrated care pathway was developed using iterative meetings with patients, informal caregivers and professionals. Current practice, barriers and incentives for change were analyzed through literature research, expert consultation, interviews, and by establishing working groups of health care professionals, patients and informal caregivers. This resulted in proposals for the improvement of the care processes, which were combined and finally resulted in the integrated care pathway.

The pathway is comprised of cross-organizational agreements on coordination and continuity of care for older patients who transfer between the hospital, the geriatric rehabilitation facility and primary aftercare in the home situation. The main components of the care pathway were the following: 1) an appointed care pathway coordinator who acted as a liaison between professionals in different organizations and encouraged communication and information exchange between the organizations involved; 2) a newly developed triage instrument was used in the hospital, which provided guidance and support in determining the eligibility of potential patients for referral to geriatric rehabilitation or to another form of rehabilitation; 3) active involvement of patients and informal caregivers in all decisions regarding their rehabilitation trajectory (in the hospital, geriatric rehabilitation facility and primary care); 4) high quality and timely submission—on the day of discharge—of all patient discharge summaries (from the hospital to the geriatric rehabilitation facility and from the geriatric rehabilitation facility to primary care providers; 5) structural (at least once or twice per year) evaluation meetings organized between professionals from the hospital, the geriatric rehabilitation facility and primary care organizations. The agreements in the integrated care pathway can be retrieved in S1 Table.

In the care as usual cohort the five aforementioned components were not established in agreements or protocols. The professionals in the care as usual cohort did not have a care pathway coordinator, or an official triage instrument. Furthermore, patients and their informal caregivers were not always involved in decisions regarding their rehabilitation trajectory. Agreements about the timeliness and quality of discharge summaries were not formally established in protocols, and there were no structural evaluation meetings between professionals of the hospital, the geriatric rehabilitation facility and primary care organizations.

### Time horizon and data collection

The costs and effects of the integrated care pathway were evaluated for every patient, over nine months, after inclusion. Because a societal perspective was used to evaluate the cost-effectiveness, intervention costs, health care costs, and patient and family costs were identified. As all participants were beyond the retirement age of 65 years, productivity losses were not taken into account in this study. Data were collected using structured face-to-face interviews with patients at baseline (at time of admission to the geriatric rehabilitation facility), after three months and after nine months. These interviews were performed by a trained research assistant. Informal caregivers of the patients received a questionnaire in which they were asked to assess the hours of informal care they provided per week. Furthermore, data were collected from the registration system in the hospital and the registration system in the geriatric rehabilitation facility.

### Costs

The intervention costs (costs of the integrated care pathway) were assessed by means of a short digital questionnaire. In this questionnaire professionals involved in the care pathway were asked to quantify the average time they had spent on tasks related to the pathway on a structural basis (e.g. costs of the care pathway coordinator and structural meetings between organizations). Costs of developing the integrated care pathway were excluded as these sunk costs will be disregarded in future implementation of the pathway. In the care as usual cohort, the intervention costs were zero.

Health care volumes were assessed by face-to-face interviews with patients. In these interviews, which were performed by a trained research assistant, participating patients were asked to indicate the healthcare services they used in a certain period (i.e. the six months before baseline, three months after baseline and six months later). The healthcare services under evaluation were temporary admission to a residential care facility, a nursing home, GP contacts, outside-of-hours GP services, home care, day care, medical specialist consultations and contact with allied health professionals, such as physiotherapists or occupational therapists. The number of days admitted to the university hospital of Maastricht and the number of days admitted to the local geriatric rehabilitation facility (part of the category ‘nursing home admissions’) were measured using registration systems from the hospital and the geriatric rehabilitation facility. Patient and family costs were also assessed in these face-to-face interviews, and can be categorized by assistive devices and environmental adaptations, hours of informal caregiving and travel expenses. Patients were asked if they purchased or received any assistive devices or environmental adaptations (e.g. in their home) and informal caregivers were asked about the number of hours per week they spent on informal care activities (i.e. domestic duties, personal care, moving outside the house and the number of hours other informal caregivers provided help). As exact travel distances to health care services were unknown, we used standard distances as recommended in the Dutch manual for cost research and reference prices in health care.[30]

Health care use, assistive devices, environmental adaptations and travel expenses were valued using the updated Dutch manual for cost research and reference prices in health care. [30] If no prices were listed in the manual (which mainly pertains to assistive devices and environmental adaptations), costs were obtained from websites specializing in the sale of assistive devices and environmental adaptations. To calculate the intervention costs, wages of professionals were multiplied by the hours they indicated spent on tasks induced by the pathway. Healthcare costs were calculated by multiplying the volume of healthcare used by the price of the unit obtained from the Dutch manual for cost research and reference prices in health care. This manual recommends to value informal care at the price of a professional housekeeper. Travel expenses were calculated by multiplying the number of visits to a healthcare service (e.g. GP contacts, medical specialist consultations and contact with allied health professionals) with standard distances and transportation prices, including parking fees. Both standard distances and transportation prices were provided by the manual for cost research and reference prices.[30]

All costs in this study were expressed in euros (€). Most of the patients were included in 2014 and therefore, all prices were adjusted by the 2014 consumer price index. Because the respondent follow-up period was nine months, discounting of effects was not needed.

### Effects

The clinical effects of this study were assessed using face-to-face interviews with patients. The primary outcome measure for this cost-effectiveness analysis (CEA) was level of dependence in activities of daily living, assessed by the KATZ Index of activities of daily living (KATZ-15).[27] This index evaluates one’s ability to perform activities of daily living using 15 questions about (instrumental) activities of daily living. Every question can be answered by ‘no help needed’ (0) or ‘help needed’ (1). The sum score ranges from 0–15 and a higher score represents more dependence in activities of daily living.[32]

The primary outcome measure for the cost-utility analysis (CUA) was quality-adjusted life years (QALY), measured with the EuroQol-5D-3L.[33] This instrument assesses one’s quality of life by measuring five domains: mobility, self-care, usual activities, pain/discomfort and anxiety/depression. The scores on these domains create a health profile, which can be converted into a utility using a tariff.[33] In this study, the Dutch tariff was used.[34] The utilities acquired at baseline, after three months and after nine months, were used to calculate QALYs using the linear area under the curve method.[35] QALYs generally range from 0–1 with a score of 1 representing a perfect health state within one year and 0 representing death. It is also possible to have a negative QALY, representing a health status ‘worse than dead’. With a follow-up period of 9 months, a minimum QALY score of -0.25 and a maximum QALY score of 0.75 could be obtained.

### Missing data

Missing data on both costs and clinical effects were assumed to be missing at random. Missing data on the costs were handled using the individual mean imputation technique. In cases where participants did not have one, single measurement of a cost item, the average of their cohort (the care as usual cohort or the care pathway cohort) was used. The mean of the group was also used for missing data on assistive devices and environmental adaptations. Missing data on the clinical effects (KATZ-15 and EQ-5D-3L) were imputed using the group mean. For patients who died, their costs and utilities were valued zero in consecutive measurement periods. Furthermore, the worst KATZ-15 score within the group the patient belonged to was taken as the KATZ-15 score for people who died.

If a patient indicated that they did not have an informal caregiver, the costs of informal caregiving were valued at zero. If the data was missing because the informal caregiver did not participate or dropped out of the study, the average cost for the group the informal caregiver belonged to (the care as usual cohort or the care pathway cohort) was used.

### Statistical analysis

Descriptive statistics, independent t-tests and Chi square tests were used to describe patients’ characteristics at baseline and to identify baseline differences between the two cohorts on the outcome measures (KATZ-15 and EQ-5D-3L). Descriptive statistics were also used to present mean volumes and costs of health care use at baseline. Due to skewedness of the cost-data, non-parametric bootstrapping (1,000 times) was performed to compare baseline costs. To correct for baseline cost differences at the patient level, a regression-based adjustment in the follow-up data was performed. This method adjusts the total costs with a regression model, where total costs are taken as the dependent variable and baseline costs as the independent variable.[36]

Costs after 9 months were compared with non-parametric bootstrapping (1,000 times). Statistically significant differences in costs were determined using a 95% Confidence Interval (CI). If the value ‘0’ was included in the CI, this was an indication of no cost difference between the groups. An incremental cost-effectiveness ratio (ICER) was calculated by dividing the difference in costs between the two cohorts by the difference in KATZ-15 score. When performing bootstrap analyses, a higher score is understood to represent a positive outcome. Therefore, only for bootstrapping purposes, the KATZ-15 scores were reversed (a higher score representing less dependence in activities of daily living).

An incremental cost-utility ratio was calculated by dividing the difference in costs by the difference in QALYs. To estimate the sample uncertainty around the ICERS, the costs and effects were also bootstrapped (5,000 times) and these 5,000 cost-effectiveness ratios and the 5,000 cost-utility ratios were presented on two incremental-cost effectiveness planes (CE-planes) with four quadrants. [33]

A cost-effectiveness acceptability curve (CEAC) was created to show the probability that the integrated care pathway is cost-effective, compared to care as usual, for a range of willingness-to-pay values. The willingness-to-pay (WTP) is the amount society is willing to pay for one extra unit of clinical effect (one QALY or one point added on the KATZ). Because the WTP threshold for the KATZ-15 is unknown, a range of values will be shown. Also, no information is available regarding the WTP for one extra QALY in our sample. The Dutch National Health Care Institute published a report in 2015 on the burden of illness and corresponding WTPs. In this report, low, moderate and high burden of illness correspond with WTPs of €20,000, €50,000 and €80,000, respectively.[37] Given the high age, frailty and multi-morbidity in our sample, we classified the participants as having a moderate burden of illness. Therefore, their corresponding WTP was €50,000. Statistical tests were performed using SPSS for Windows version 22.0 and bootstrapping was done using Excel 2010.

### Sensitivity analyses

Five sensitivity analyses were conducted to evaluate the robustness of the results: 1) taking only survivors into account; 2) taking only complete cases into account; 3) using a different KATZ-15 score for patients who died; 4) using the healthcare perspective, and 5) using QALYs based on the UK tariff instead of the Dutch tariff. First, due to the frailty level of the population, a large percentage of patients dropped out during the course of the study. Therefore, a large part of the data was imputed using individual mean imputation (costs), mean imputation (clinical effects) or valuing costs and utilities at zero and using the worst KATZ-15 score of the cohort in consecutive measurement periods (patients who died). To investigate the potential impact of imputing this data, the first sensitivity analysis only took survivors into account and the second analysis was performed with only complete cases. The third sensitivity analysis used a KATZ-15 score of 15 (total dependence in activities of daily living) as a score for patients who died, instead of the worst KATZ-15 score of the cohort. Furthermore, because the intervention costs were roughly estimated and because possible (monetary) gains (caused by increased efficiency incurred by the pathway) were not measured, the societal perspective for calculating costs was compared with a health care perspective. Finally, as utilities can be calculated using different tariffs, the last sensitivity analysis was performed with QALYs based on the UK tariff.

## Results

### Study population

In total, 49 patients in the care as usual cohort agreed to participate in the study (69% of the eligible 71 patients) and 113 patients were included in the care pathway cohort (60% of the eligible 189).

**Fig 1:**
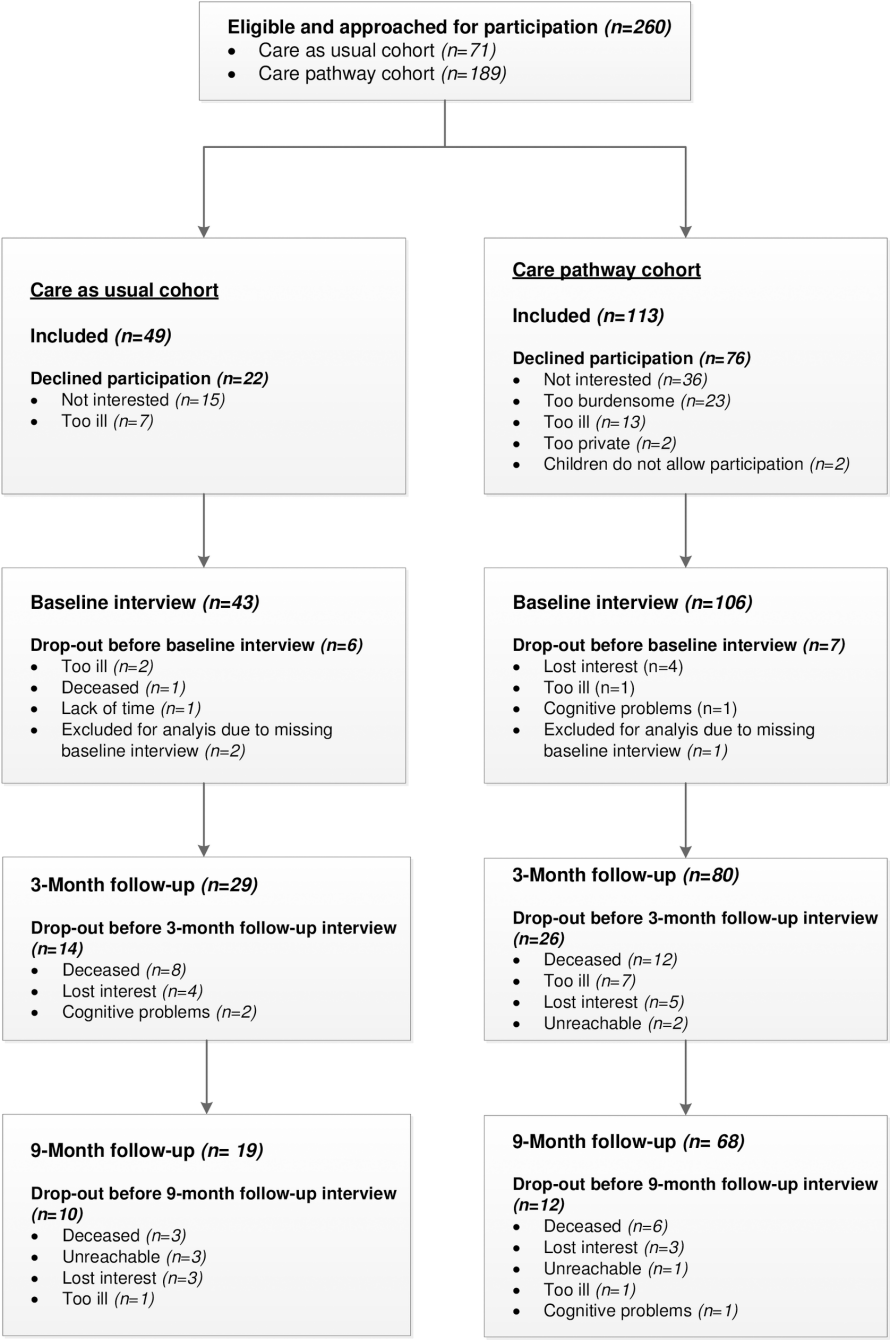
Flowchart of patients.

Prior to the baseline interview, 6 patients in the care as usual cohort and 7 patients in the care pathway cohort dropped out of the study due to various reasons. Therefore, a total of 43 and 106 participants were included in the study. Fig 1 shows the flowchart of the patients in the study and the reasons for drop-out. Fig 1 also shows that the percentage of missing data after three months was 32.6% (n = 14) in the care as usual cohort and 60.5% (n = 26) in the care pathway cohort. These percentages were 22.6% (n = 24) and 35.8% (n = 38), respectively, after nine months.

In the care as usual cohort, 26 informal caregivers were included. Out of the included 43 patients, 9 patients (20.9%) did not have an informal caregiver and 8 informal caregivers did not want to participate because they were not interested (n = 4) or the person they cared for had died (n = 4). In the care pathway cohort, 28 informal caregivers were included. 32 out of 106 patients (30.2%) indicated they did not have an informal caregiver. The two other main reasons for not participating were no interest in participation (n = 24), and patients did not want to burden their informal caregiver (n = 10).

As shown in Table 1, background characteristics measured at baseline are comparable for the patients in the two cohorts.

**Table 1:** Background characteristics of patients in both cohorts measured at baseline.

	Care as usual cohort n = 43	Care pathway cohort n = 106	p-value
**Characteristics**			
Mean age (sd)	79.6 (7.1)	80.7 (6.9)	0.370
Sex (% female)	65.0%	67.9%	0.471
Living situation (% living alone)	67.4%	68.9%	0.865
Education (% High education)	60.5%	67.9%	0.385
Mean number of morbidities (sd)	3.6 (2.1)	3.5 (1.8)	0.882
**Outcome measures**			
KATZ-15 mean score (sd) (range 0–15*)	6.6 (3.6)	5.7 (3.3)	0.156
EQ-5D-3L Dutch tariff mean score (sd) (range -0.329–1*)	0.53 (0.28)	0.51 (0.30)	0.622

*The underlined score represents the most preferable score.

Health care use and patient and family costs at baseline are displayed in Table 2. This table shows that at baseline there was a significant difference in total health care costs between the two cohorts. This difference can be explained by the fact that, patients in the care as usual cohort spent significantly more days in the hospital in the last six months compared to patients in the care pathway cohort (8.8 days versus 3.5 days). Furthermore, patients in the care pathway cohort spent significantly less days at day care compared to patients in the care as usual cohort (0 versus 9.1). Patient and family costs did not significantly differ between the cohorts at baseline. The total baseline costs were significantly higher in the care as usual cohort (€13,777 versus €10,311). For this reason, a regression-based adjustment was performed.

**Table 2:** Healthcare use/costs, patient and family costs at baseline.

	Care as usual cohort (n = 43)		Care pathway cohort (n = 106)		Bootstrapping
Healthcare use/costs (last 6 months)	Mean use (SD)	Total costs (€)	Mean use (SD)	Total costs (€)	95% CI
Days in hospital	8.8 (14.3)	5,600.6 (1351.7)	3.5 (7.7)	2,180.6 (470.5)	(-6,519, -789)*
Days in nursing home	2.1 (14.0)	672.2 (654.4)	0.3 (3.3)	103.6 (100.6)	(-1,997, 299)
Days in care home	0.0 (0.0)	0 (0)	9.1 (39.1)	898.1 (381.6)	(210, 1721)*
Regular contact with GP	2.9 (2.6)	94.5 (13.4)	4.1 (4.8)	134.6 (15.3)	(-1, 80)
Contact with GP during out-of-office hours	0.4 (0.7)	45.7 (13.4)	0.6 (1.2)	70.1 (13.9)	(-12, 62)
Professional homecare (hours/week)					
• Nursing care	0.2 (0.6)	299.4 (160.0)	0.04 (0.3)	98.4 (55.7)	(-567, 92)
• Personal care	1.0 (1.8)	1,354.2 (337.9)	1.1 (1.8)	1,392.2 (222.8)	(-746, 804)
• Domestic care	1.6 (1.7)	835.7 (138.0)	1.3 (1.4)	653.6 (69.5)	(-489, 102)
Number of half days per week in day care	0.2 (0.7)	325.6 (184.3)	0.05 (0.4)	83.2 (68.1)	(-656, 76)
Contact with medical specialist	2.2 (2.9)	114.4 (22.0)	2.4 (2.6)	125.1 (13.2)	(-39, 60)
Contact with allied professional	8.5 (18.1)	281.0 (88.7)	11.5 (15.9)	394.3 (56.1)	(-89, 320)
Total healthcare costs		9,604.6 (1620.1)		6,096.0 (667.5)	(-6,884, -246)*
**Patient and family costs (last 6 months)**					
Costs assistive devices/environmental adaptations		371.7 (107.6)		410.7 (94.8)	(-245, 301)
Total travel costs		202.9 (2.2)		204.1 (1.2)	(-4, 6)
Informal care (hours per week)	10.1 (12.8)	3,658.4 (687.3)	9.8 (9.0)	3,559.8 (313.1)	(-1,677, 1224)
**Total costs**		13,777.1 (1639.0)		10,310.8 (804.3)	(-7,177, -198)*

*Statistically significant difference

### Cost analysis

Intervention costs of the integrated care pathway were on average, €77.60 per patient. These costs consisted mainly of the care pathway coordinator and the structural evaluation meetings. Total societal costs during the nine month follow-up period for the care as usual cohort were €62,170, on average, whereas for the care pathway cohort, they were €50,791. These total costs were adjusted for baseline cost differences using the regression-based adjustment method.[36] As shown by the confidence interval, this difference is statistically significant (CI -22,090, -988). These lower costs are mainly the result of shorter hospital stays (39.2 vs. 27.0 days) and shorter stays in the geriatric rehabilitation facility (79.1 vs. 55.4 days) (Table 3). This table also shows that the number of contacts with the GP increased in the care pathway cohort (3.3 visits for the care as usual cohort versus 4.9 visits for the care pathway cohort; CI = 11, 98) and that the number of visits to a day care center significantly decreased (on average, 0.5 half days per week in the care as usual cohort and 0.1 half days per week in the care pathway cohort; CI = 1,576, 40). The total healthcare costs in the care pathway cohort were also significantly lower (57,350 vs. 42,516; CI = -24,900, -4,525). Patient and family costs did not significantly differ between the cohorts.

**Table 3:** Healthcare use/costs, patient and family costs during the nine month follow-up period.

	Care as usual cohort (n = 43)		Care pathway cohort (n = 106)		Bootstrapping
Healthcare costs	Mean use (SD)	Total costs (€)	Mean use (SD)	Total costs (€)	95% CI
Intervention costs		0		77.6	
Days in hospital	39.2 (21.4)	20,861.5 (1808.1)	27.0 (26.3)	13,555.7 (1124.0)	(-11,358, -3,310)*
Days in nursing home	79.1 (75.8)	24,902.4 (3651.4)	55.4 (38.5)	17,229.0 (1211)	(-15,613, -252)*
Days in care home	13.0 (58.1)	1,205.6 (841.3)	13.6 (55.4)	1,355.3 (548.1)	(-1,804, 2,012)
Regular contact with GP	3.3 (3.1)	109.1 (14.8)	4.9 (5.0)	162.0 (16.2)	(11, 98)*
Contact with GP during out-of-office hours	0.3 (0.5)	32.6 (54.9)	0.5 (1.4)	8.4 (15.2)	(-9, 58)
Professional homecare (hours/week)					
• Nursing care	0.5 (1.8)	617.5 (664.1)	0.4 (1.3)	368.2 (206.2)	(-888, 761)
• Personal care	2.7 (3.5)	2,721.2 (534.4)	3.4 (4.2)	3,404.5 (406.5)	(-659, 2029)
• Domestic care	3.0 (4.0)	1,227.4 (236.1)	3.0 (3.2)	1,193.1 (124.9)	(-593, 443)
Number of half days per week in day care	0.5 (1.5)	815.7 (410.8)	0.1 (0.5)	137.5 (76.6)	(1,576, 40)*
Contact with medical specialist	3.2 (3.6)	167.6 (27.7)	4.5 (4.5)	236.4 (21.8)	(-3, 132)
Contact with allied professional	16.7 (29.2)	542.1 (139.7)	27.4 (29.7)	897.9 (94.2)	(-7, 676)
Total healthcare costs		57,350.1		42,516.4	(-24,900, -4,525)*
**Patient and family costs**					
Costs assistive devices/environmental adaptations		647 (181.5)		588.7 (101.7)	(-489, 312)
Total travel costs		400.2 (2.7)		406.8 (2.3)	(-1, 13)
Informal care (hours per week)	27.0 (36.5)	7,701.2 (1730.4)	20.3 (23.0)	5,762.6 (657.7)	(-5,900, 1,302)
**Total costs unadjusted**		65,993.19 (4732.0)		49,232.4 (2467.6)	(-27,248, -6,721)*
**Total costs∞**		62,169.59 (4807.91)		50,791.38 (2473.7)	(-22,090, -988)*

*Statistically significant difference

**∞** Adjusted for baseline differences.

### Cost-effectiveness and cost-utility

Table 4 shows the incremental cost-effectiveness and the incremental cost-utility. Implementation of the integrated care pathway resulted in less dependence in activities of daily living (1.04) and lower costs (-€11,605). The difference in QALYs between the two groups was 0.01.

**Table 4:** Differences in costs and effects between the two cohorts and corresponding ICERS.

		Total costs in €			Total effects*			
Analysis	Effect measure	CUC (n = 43)	CPC (n = 106)	Δ Costs	CUC (n = 43)	CPC (n = 106)	Δ Effects	ICER
Cost-effectiveness	KATZ-15	62,326	50,720	-11,605	8.52	9.56	1.04	-11,186
Cost-utility	QALY	62,326	50,720	-11,605	0.37	0.377	0.01	-2,304,876

CUC = Care as Usual Cohort; CPC = Care Pathway Cohort; ICER = Incremental Cost-Effectiveness Ratio.

* For bootstrapping purposes, the KATZ-15 scores have been reversed; therefore a higher score represents less dependence in activities of daily living.

**Fig 2:**
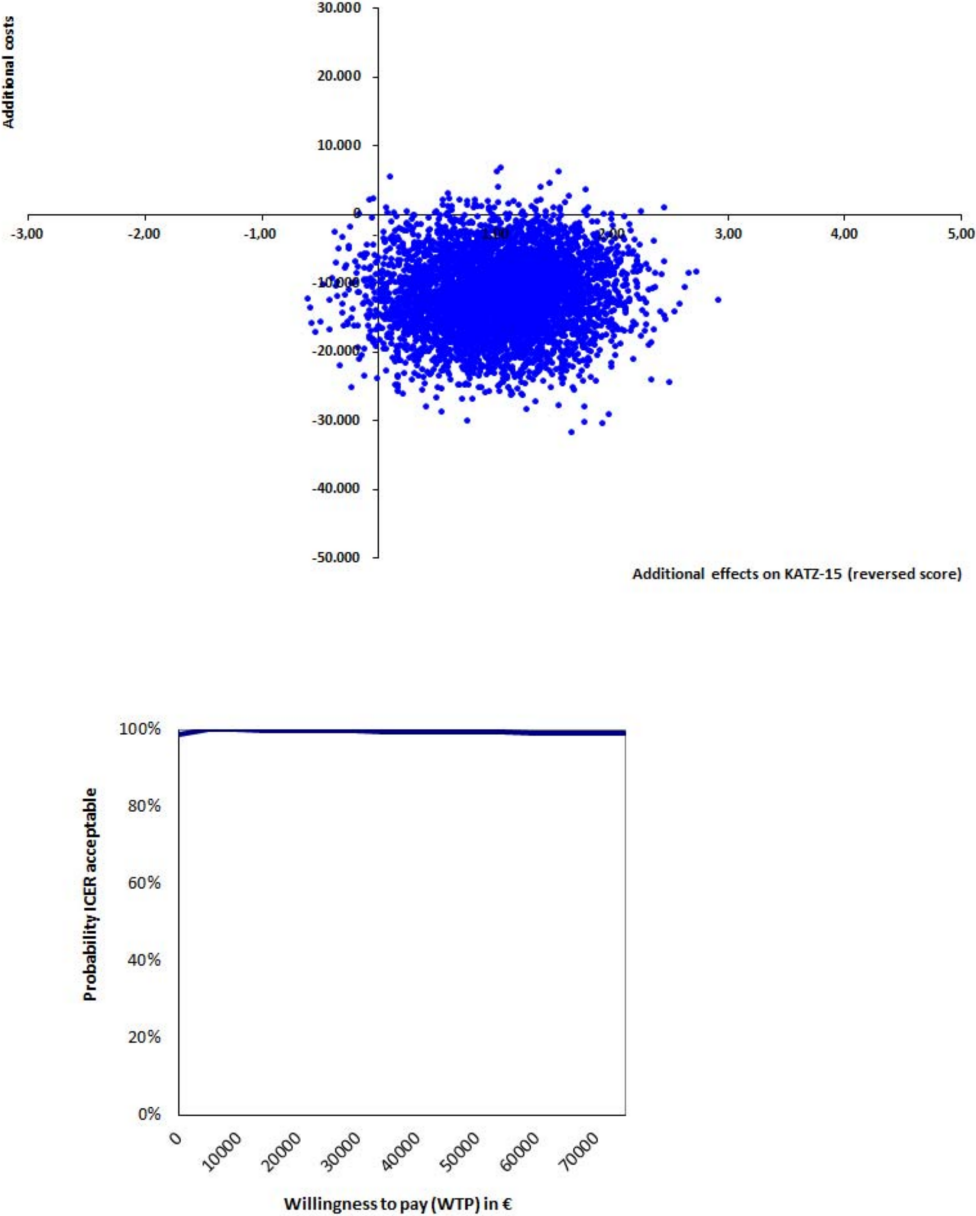
Cost-effectiveness plane and incremental cost-effectiveness acceptability curve KATZ-15.

As displayed in the cost-effectiveness plane for the KATZ-15 (Fig 2), 97% of the bootstrapped ICERS were in the dominant (southeast) quadrant, indicating more effects and lower costs. As the willingness-to-pay threshold for daily functioning as measured with KATZ-15 is unknown, a range of WTP thresholds are shown in the cost-effectiveness acceptability curve in Fig 2 (see Methods). As the pathway results in more effects and saves costs, this curve shows that the probability of the integrated care pathway being cost-effective (when compared to care as usual) remains 99% or higher for a range of willingness-to-pays.

**Fig 3:**
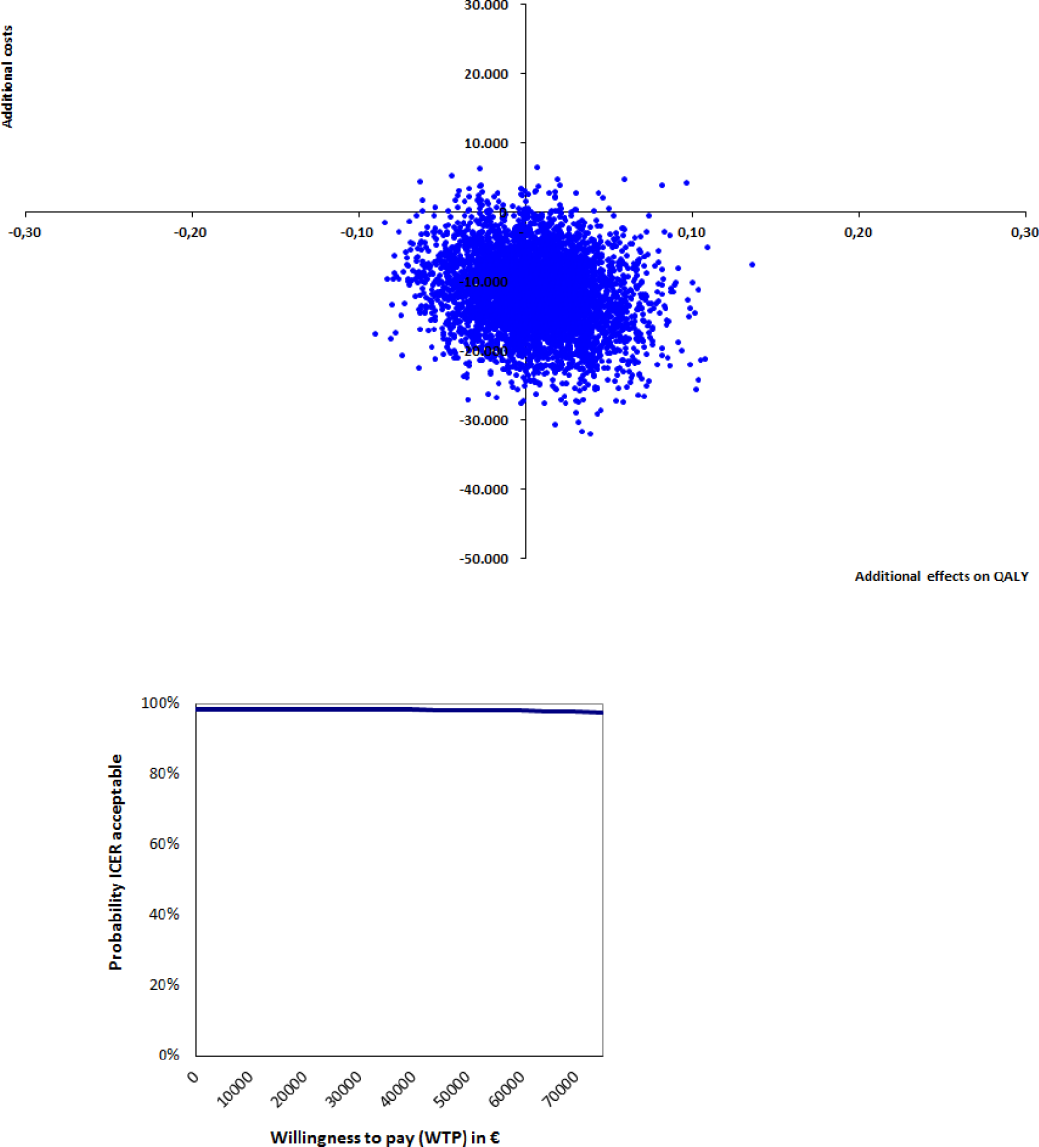
Cost-utility plane and incremental cost-utility acceptability curve QALY.

The cost-utility plane for QALYS (Fig 3) displays that 56% of the incremental cost-utility ratios were located in the dominant quadrant. Due to the fact that no differences in QALYs were detected, all remaining ratios were in the southwest quadrant. As shown by the CEAC in Fig 3, the probability of the integrated care pathway being cost-effective, compared to care as usual at WTP of €50.000 (moderate burden of illness), is 98%.

### Sensitivity analyses

**Fig 4:**
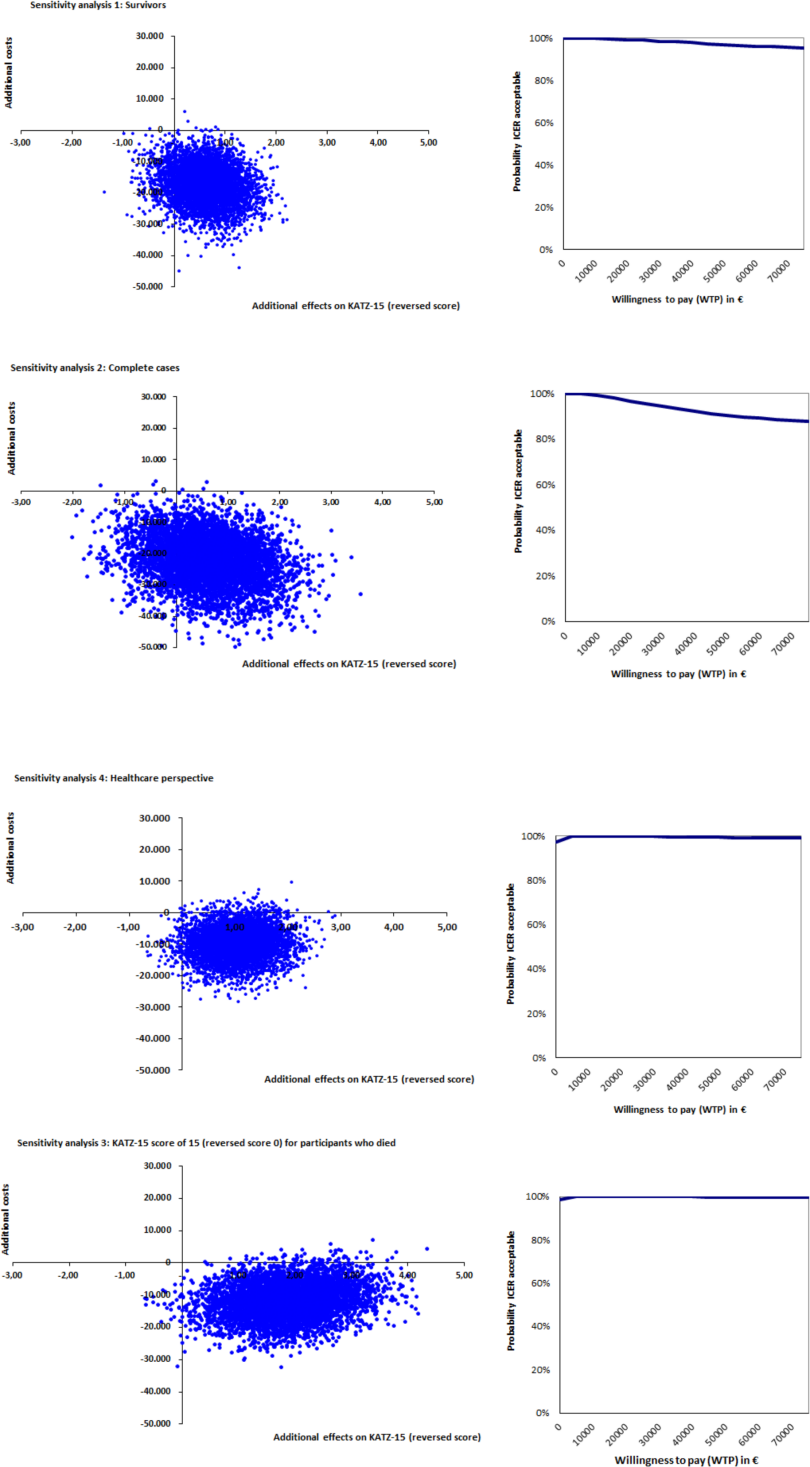
Sensitivity analyses KATZ-15.

The sensitivity analyses show fair robustness of the results for all cost-effectiveness analyses (Table 5). Where 97% of all ICERs were located in the dominant quadrant, this percentage ranges from 78% to 100% in the five sensitivity analyses. As shown in Fig 4, the probability of the pathway being cost-effective remains high and stable for a range of WTPs in all sensitivity analyses.

**Table 5:** Results of the base case analysis and the sensitivity analyses.

				Distribution (%) of ICERS in cost-effectiveness plane			
	Δ Costs	Δ Effects	ICER*	NE*	SE* (dominant)	SW*	NW* (inferior)
Base case analysis; CUC* *(n = 43)*, CPC* *(n = 106)*							
KATZ-15	-11,605	1.04	-11,186	1%	97%	2%	0%
QALY	-11,605	0.01	-2,304,876	0%	56%	43%	1%
*Sensitivity analysis 1*: *Only survivors; CUC (n = 32)*, *CPC (n = 88)*							
KATZ-15	-17,139	0.62	-27,724	0%	90%	10%	0%
QALY	-17,139	-0.02	1,100,879	0%	34%	66%	0%
*Sensitivity analysis 2*: *Complete cases; CUC (n = 19)*, *CPC (n = 68)*							
KATZ-15	-22,298	0.62	-36,101	0%	79%	21%	0%
QALY	-22,298	-0.01	3,397,262	0%	43%	57%	0%
*Sensitivity analysis 3*: *KATZ-15 score of 0 for participants who died; CUC (n = 43)*, *CPC (n = 106)*							
KATZ-15	-11,605.3	1.87	-6,191	1%	98%	1%	0%
*Sensitivity analysis 4*: *Healthcare perspective; CUC (n = 43)*, *CPC (n = 106)*							
KATZ-15	-9,693	1.04	-9,342	3%	95%	2%	0%
QALY	-9,693	0.01	-1,925,041	1%	55%	43%	1%
*Sensitivity analysis 5*: *QALY UK Tariff CAU (n = 43)*, *CPC (n = 106)*							
QALY	11,605	-0.02	661,873	0%	22%	76%	2%

*CUC = Care as Usual Cohort; CPC = Care Pathway Cohort; ICER = Incremental Cost-Effectiveness Ratio, NE = north-east quadrant; SE = south-east quadrant; SW = south-west quadrant; NW = north-west quadrant

**Fig 5:**
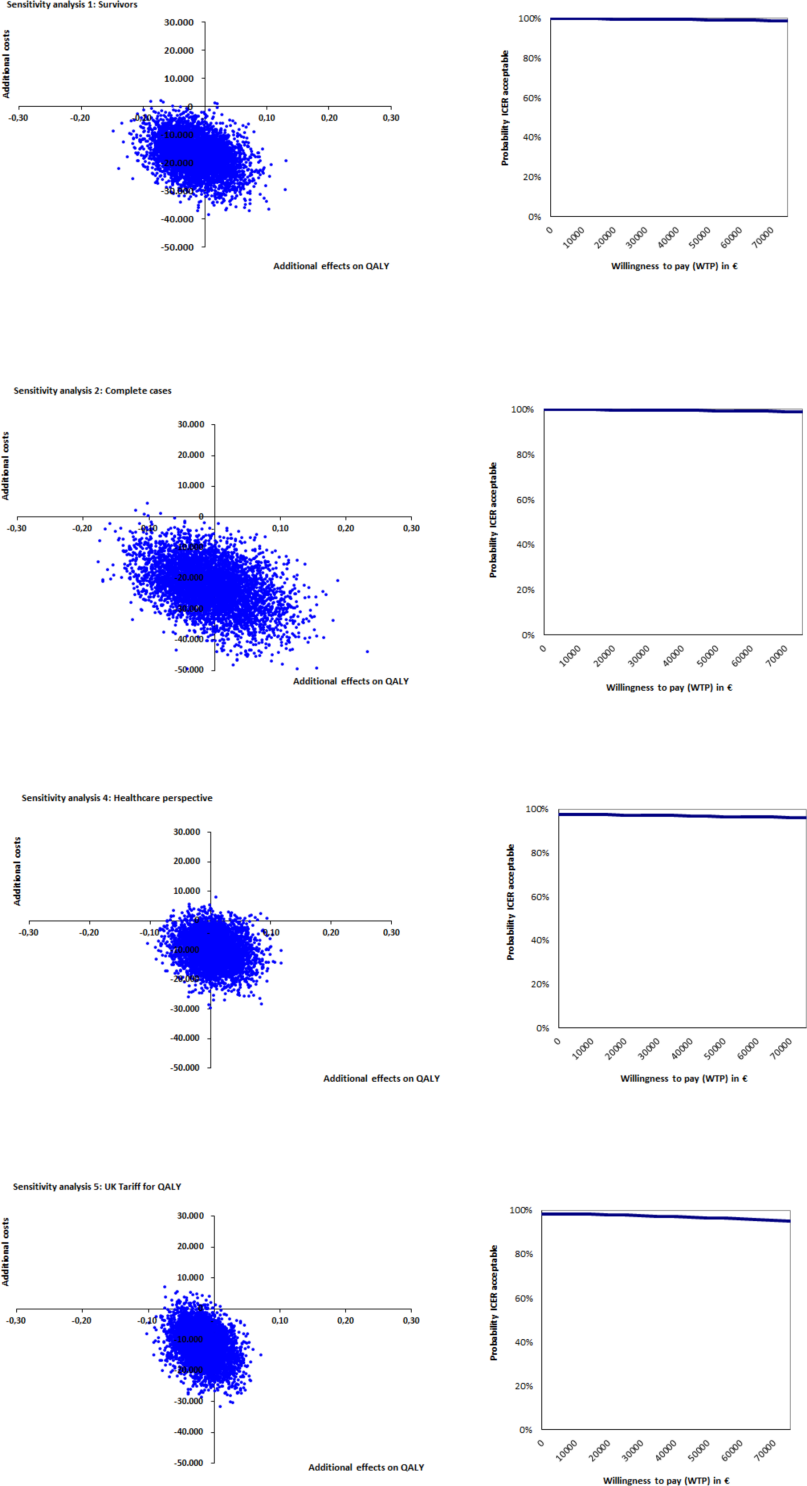
Sensitivity analyses QALY.

When looking at the sensitivity analyses for QALYs, all show no effects and a large decrease in costs (Table 5 and Fig 5). In the base case analysis, 56% of the bootstrapped incremental cost-utility ratios were in the dominant quadrant. In the four sensitivity analyses, the percentage of bootstrapped incremental cost-utility analyses located in the dominant quadrant ranges from 22% to 55%. Sensitivity analysis 5 (UK tariff for QALYs) causes the largest shift of ICERs towards the south-west quadrant.

## Discussion

The results of this study indicate that the integrated care pathway is a cost-effective alternative, compared to care as usual, on dependence in activities of daily living (measured with the KATZ-15). The cost-effectiveness pane shows that 97% of all bootstrapped ICERs were located in the dominant quadrant. Although no WTP threshold for dependence in activities of daily living exists, the cost-effectiveness acceptability curve for the KATZ-15 indicates that the integrated care pathway is a cost-effective intervention. Sensitivity analyses show robustness of results for this outcome measure: when only survivors or complete cases were taken into account, when changing the score on the KATZ-15 for patients who died, and when data was analyzed from a healthcare perspective, the pathway remained a cost-effective intervention compared to care as usual.

With no effects but large cost savings on the outcome measure QALY, almost all bootstrapped ICERs were located south of the x-axis in the cost-effectiveness pane. When using a WTP threshold of €50,000 (moderate burden of illness), the probability of the pathway being cost-effective was 98%. The sensitivity analysis which analyzed costs from a healthcare perspective showed roughly the same results as the base case analysis, whereas the other three sensitivity analyses (survivors, complete cases and the UK tariff for QALYs), all resulted in a shift of the bootstrapped ICERs from the dominant quadrant towards the south-west quadrant.

Thus, both cost-effectiveness analyses and cost-utility analyses show a large cost decrease when comparing the care as usual cohort with the care pathway cohort. This cost decrease was mainly caused by a decrease in health care costs related to hospital stays and stays in the geriatric rehabilitation facility. This might be an indication that due to implementation of the integrated care pathway, the possibility for timely transfer of patients to the next setting, improved. Also, the use of the triage instrument helped distinguish patients who were eligible for geriatric rehabilitation from patients who were best suited for another type of care. This may also have resulted in an improved patient flow throughout the trajectory. The decreased length of stay in hospital and geriatric rehabilitation facility did not coincide with a significant difference between patient and family costs for the two cohorts. This might indicate that the care burden for the family caregivers did not rise as a consequence of this decreased length of stay.

Although implementation of the pathway resulted in less dependence in activities of daily living among patients, the effect on QALYs measured with the EQ-5D-3L was 0.01 in the base case analysis and ranged from -0.02 to 0.01 in the four sensitivity analyses. It can thus be concluded that implementation of the pathway did not affect quality adjusted life years among patients. A likely explanation for this lack of effect is that therapy in the geriatric rehabilitation facility is mostly directed towards regaining functional status, such as independence in (I)ADL activities and mobility.[38, 39] This means that patients are being trained to safely return home and once this goal has been reached, they will be discharged from the geriatric rehabilitation facility. Less attention is being paid to improving other domains of quality of life included in the EQ-5D-3L, such as mood. Also, training older adults to restart social activities or other hobbies once discharged and returned home, is not regarded as a main goal of geriatric rehabilitation, though it is likely to influence overall quality of life. A last explanation for the lack of effects on QALYs is that a process evaluation conducted alongside this study (described elsewhere [40]) showed that not all five key pathway components were fully implemented according to plan. This indicates that there is still room for improvement, for instance in the provision of information to patients and their informal caregivers, and in the quality and timing of medical discharge summaries.

Our study is the first to perform a thorough economic evaluation of an integrated care pathway in geriatric rehabilitation from a societal perspective, and to take into account the costs incurred in three different settings (hospital, geriatric rehabilitation facility and primary care). As previously mentioned, literature on studies analyzing the cost-effectiveness of (intersectoral) integrated care pathways is scarce.[24] The few studies that have assessed the clinical effects of care pathways, in terms of costs, usually reveal a decrease in costs due to shorter hospital length of stay, which is in accordance with our results.[21, 26, 28, 41] Still, it is not possible to compare our results to these studies as they vary in perspective (healthcare perspective or hospital perspective instead of societal perspective), patient groups and settings. Furthermore, the methodological quality of these studies was often poor and the calculation of costs, not always described.[21, 41]. According to Nolte and Pitchforth there is some evidence of cost-effectiveness of (intersectoral) integrated care approaches but the evidence remains weak [23]. It is therefore important that performing methodologically sound economical evaluations should be part of a comprehensive evaluation framework that examines the efficiency and efficacy of integrated care pathways.

### Generalizability of results

As the organizations involved in our study are fairly representative of the Dutch situation, we expect that our findings are applicable to other health care facilities throughout the Netherlands. Therefore, we believe that broader implementation of the integrated care pathway in the Netherlands could result in cost-savings on a wider scale. Because health care systems and patient populations differ across countries, the effects might not be directly transferable to other countries. Still, many countries recognize problems in continuity and coordination of care among older adults experiencing similar care trajectories. Therefore, elements of this integrated care pathway, such as inter-organizational collaboration and communication between providers, may be relevant outside the Netherlands as well. However, it is important for organizations to adapt the content of this integrated care pathway to local needs and settings with the help of end-users of the pathway.

### Strengths and limitations

This study is subject to several limitations. First, the two cohorts were studied during different periods (the care as usual cohort in 2011–2012 and the care pathway cohort in 2013–2014). The reason for this is that we wanted to perform our research in one care setting, so that the two groups are highly comparable with regard to setting related factors. However, we cannot completely rule out that time related factors have influenced the results. Furthermore, the use of the triage instrument, a key component of the integrated care pathway, imposed stricter admission rules for geriatric rehabilitation. This influenced the type of patients who were eligible for geriatric rehabilitation. These stricter admission rules could be an explanation for the difference in baseline costs between the two cohorts. However, because we adjusted for this baseline costs difference using a regression-based method, and because there were no differences in baseline characteristics, we believe that this potential selection bias was sufficiently accounted for. Third, due to the frailty of our population, a large percentage of patients dropped out during the course of our study, and therefore, a substantial amount of data was imputed. As imputing data is subject to assumption, this might have caused bias. To minimize this bias, we used the most preferred method for handling missing data, which is mean imputation for the outcome measures KATZ-15 and QALYs and individual mean imputation to impute costs.[42] Furthermore, sensitivity analyses without imputed data showed fairly similar results, demonstrating that the results are robust. Fourth, healthcare costs (except hospital admissions and admissions to the geriatric rehabilitation facility), costs of assistive devices and environmental adaptations and hours of informal care were estimated based on the self-reporting of patients and informal caregivers. As self-reported measures are always susceptible to recall bias, this might have influenced the results.[43] Nevertheless, we believe that recall bias was equally present in both cohorts. Finally, to assess the intervention costs, we asked professionals to indicate the number of hours they had spent on tasks related to the integrated care pathway. However, these tasks might not be easily isolated from usual care practice. Therefore, the intervention costs might be underestimated. Because the intervention costs are low compared to the total costs (€77.60 per patient compared to the total costs of €50,791) there is little chance this could not have influenced the results.

A strength of this study is that thorough research into the cost-effectiveness of integrated care pathways is scarce, in particular, in the cost-effectiveness of care pathways crossing organizational borders. Therefore, the result of this study adds new evidence to the complex field of integrated care pathways and geriatric rehabilitation. Another strength lies in the fact that this study is performed from a societal perspective, including longitudinal observations, providing a complete view of all costs and effects.

## Conclusion

From the current study it can be concluded that the integrated care pathway is a cost-effective intervention compared to care as usual. The integrated care pathway resulted in less dependence in activities of daily living and in fewer costs, illustrated by the fact that 97% of all bootstrapped ICERs were located in the dominant quadrant. As no effects were found on QALYs, 58% of all ICERs were located in the dominant quadrant and 43% in the south-west quadrant. Still, when using a WTP threshold of €50.000 per QALY, there is a 98% chance that the integrated care pathway is a cost-effective intervention when compared to care as usual. Based on these results, dissemination of the integrated care pathway on a wider scale could be considered. This would provide us the opportunity to confirm the findings of our study in larger economic evaluations. Furthermore, to improve the effects on QALYs, we advise to explore if therapy in geriatric rehabilitation could also focus on improving other quality of life-related domains, such as mood and social participation.

## Supporting information

S1 Table Innovative integrated care pathway for geriatric rehabilitation. (DOCX)
