# Supplementary material for: An economic evaluation of an integrated care pathway in geriatric rehabilitation for older patients with complex health problems
Source: PLoS One. 2018 Feb 28;13(2):e0191851. doi: 10.1371/journal.pone.0191851 (PMC5830039; doi:10.1371/journal.pone.0191851)
Supplement: S1 Table — (DOCX) [file pone.0191851.s001.docx]

**S1 Table.** Innovative integrated care pathway for geriatric rehabilitation.

| Setting | No. | Care pathway element |
| --- | --- | --- |
| Hospital | 1 | If the main treatment provider believes that the patient is eligible for geriatric rehabilitation, the discharge nurses of the hospital will be consulted. Preferably, this consultation takes place well in advance of discharge. |
|  | 2 | Dismissal from the hospital is preceded by a triage by a discharge nurse. Information about the patient's functional prognosis, endurability, teachability and trainability and the patient’s and informal caregiver’s needs and abilities needs to be gathered to make this triage decision. |
|  | 3 | The triage is always performed under the responsibility of an elderly care physician from the geriatric rehabilitation facility. If the discharge nurse has doubts about eligibility of the patient for geriatric rehabilitation, the elderly care physician should be consulted. |
|  | 4 | Information about functional prognosis, endurability, teachability and trainability and needs and abilities of the patient should be gathered by consulting professionals in the hospital who have been involved in the patient’s care. |
|  | 5 | The patient should always be asked about their needs and abilities and this should explicitly be taken into account when making the triage decision. |
|  | 6 | The informal caregiver should (if applicable) be asked about their ability to provide informal care and this should explicitly be taken into account when making the triage decision. |
|  | 7 | The discharge nurse should always provide oral and written information about geriatric rehabilitation to the patient and the informal caregiver. |
|  | 8 | On the day the patient is discharged from the hospital, an up-to-date list of medications, a medical and nursing discharge summary and, if necessary, a discharge summary from allied health professionals should be available for the professionals in the geriatric rehabilitation facility. |
| Geriatric rehabilitation facility | 9 | In the cases where the patient discharge summaries are not available on the day the patient is admitted to the geriatric rehabilitation facility, professionals from the geriatric rehabilitation facility should contact the hospital directly. |
|  | 10 | All patients with complex care needs admitted to the geriatric rehabilitation facility receive a systematic and multidisciplinary examination to determine which rehabilitation program is suitable for the patient. |
|  | 11 | The patient’s rehabilitation program will be established in close consultation with patient and (if applicable) informal caregiver. The patient’s wishes and abilities and their informal caregiving situation will be taken into account when determining this program. |
|  | 12 | Multidisciplinary meetings are organized at least twice during the patient´s stay. |
|  | 13 | Patients and (if applicable) informal caregivers should always receive feedback on the issues discussed during the multidisciplinary meetings. In those cases where a modification to the patient’s rehabilitation program is desirable, this will be discussed with the patient and informal caregiver. |
|  | 14 | Within two weeks after admission to the geriatric rehabilitation facility, the patient and (if applicable) informal caregiver will be informed about the patient’s provisional discharge date. |
|  | 15 | The treatment intensity should be adjusted (decreased or increased) if this is required by the progress the patient is making. |
|  | 16 | The provisional discharge date should be adjusted (decreased or increased) if this is required by the progress the patient is making. |
|  | 17 | Well before discharge, the patient’s home situation should be mapped out by a physiotherapist or occupational therapist. |
|  | 18 | After the home visit, advice should be given to the patient about required adjustments and assistive devices in the home. |
|  | 19 | The nurses in the geriatric rehabilitation facility should arrange home care prior to discharge of the patient. |
|  | 20 | If the situation of the patient is complex, a professional of the home care organization will visit the geriatric rehabilitation facility for an intake. |
|  | 21 | A professional of the home care organization will visit the geriatric rehabilitation facility for an intake if this is preferred by the patient. |
|  | 22 | An up-to-date nursing discharge summary will be sent to the home care organization on the day of discharge. |
|  | 23 | An up-to-date prescription for medication will be sent to the patient’s pharmacy on the day of discharge. |
|  | 24 | An up-to-date discharge summary by allied health professionals will be given to the patient on the day of discharge. |
|  | 25 | An up-to-date medical discharge summary and medication list will be sent to the patient’s general practitioner on the day of discharge. |
|  | 26 | The discharge summary to the general practitioner includes information on the follow-up care advised. |
| Primary care | 27 | In those cases where the patient discharge summaries are not available to primary care on the day the patient is discharged from the geriatric rehabilitation facility, professionals from the primary care organizations should directly contact the geriatric rehabilitation facility. |
|  | 28 | Once the patient is discharged from the geriatric rehabilitation facility, the nurse practitioner or district nurse in primary care should act as the patient’s case manager. |
| All settings | 29 | A care pathway coordinator is appointed. The role of the care pathway coordinator is to act as a port of call for professionals involved in the pathway, to improve communication between professionals from different settings, improve continuity and coordinate care and to further streamline the pathway. |
|  | 30 | At least twice per year, a meeting is organized between professionals from the hospital and from the geriatric rehabilitation facility who are involved in the triage process. The aim of this meeting is to evaluate whether or not the triage process, the medical discharge summaries and the transfer of patients between the hospital and the geriatric rehabilitation facility are satisfactory. |
|  | 31 | At least once a year a meeting is organized between professionals from the geriatric rehabilitation facility and from primary care to evaluate the timing and quality of the medical discharge summaries and patient transfers. |
